# Long-Term Follow-Up of the Reliability, Accuracy, and Morbidity of Dynamic Sentinel Lymph Node Biopsy in Patients with Intermediate- and High-Risk Penile Cancer and Clinically Negative Lymph Node Status

**DOI:** 10.3390/jcm15166449

**Published:** 2026-08-20

**Authors:** Ákos Pytel, Bence Pytel, Dávid Semjén, Zsombor Ritter

**Affiliations:** 1 Department of Urology, Medical School, University of Pécs, Munkacsy Mihaly u. 2., H-7621 Pecs, Hungary; 2 Department of Anatomy, Medical School, University of Pécs, Szigeti u. 12., H-7624 Pecs, Hungary; pytel.bence@aok.pte.hu; 3 Department of Pathology, Medical School, University of Pécs, Szigeti u. 12., H-7624 Pecs, Hungary; semjen.david@pte.hu; 4 Department of Medical Imaging, Division of Nuclear Medicine, Medical School, University of Pécs, Ifjusag u. 13., H-7624 Pecs, Hungary; ritter.zsombor@pte.hu

**Keywords:** penile cancer, sentinel lymph node, dynamic sentinel lymph node biopsy

## Abstract

**Objectives**: The aim of this study was to evaluate the reliability, accuracy, and morbidity of dynamic sentinel lymph node biopsy (DSNB) in patients with intermediate- and high-risk penile cancer who had clinically negative lymph nodes, with a focus on long-term follow-up outcomes. **Methods**: Between January 2005 and January 2024, 287 patients with histologically confirmed penile cancer underwent radioisotope-guided DSNB at the Department of Urology, University of Pécs. Patients lost to follow-up within 24 months were excluded. Of the remaining patients, 211 underwent primary DSNB and 37 secondary DSNB. Thirty-nine patients in the primary DSNB group with Tis, T1a, or G1 tumours were additionally excluded, leaving 209 patients for analysis. Variables included tumour stage and grade, sentinel lymph node (SLN) yield and histology, complications, inguinal progression, false-negative rate, and radical lymphadenectomy findings. **Results**: A total of 573 SLNs were removed from 209 patients, with a median of 2.7 nodes per patient. SLN metastases were identified in 31 patients, involving 37 groins. Among 178 patients with negative SLNs, three developed inguinal metastases. Sensitivity was 91% per patient and 92.5% per groin, with false-negative rates of 8% and 7.5%, respectively. All false-negative cases occurred within two years, with no later inguinal recurrences. DSNB-related complications occurred in 24 patients (11.4%); most were mild or moderate and managed on an outpatient basis. Two patients required surgery and one required hospital readmission. **Conclusions**: In a high-volume centre, DSNB is a safe and reliable staging procedure with low morbidity in clinically node-negative intermediate- and high-risk penile cancer. Further studies should assess radiotracer injection sites and compare one-day versus two-day protocols.

## 1. Introduction

Penile cancer is one of the rarest malignancies due to its low incidence rate. Based on the world standard population, the estimated incidence is 0.84 cases per 100,000 person-years [1]. The most important prognostic factor for survival is the lymph node status of the patient. In patients with clinically positive lymph node status, the treatment strategy is straightforward, and radical inguinal lymphadenectomy is indicated [2].

However, the management of patients with clinically negative lymph node status remains more controversial. Since current imaging modalities are unable to reliably detect micrometastases, the European Association of Urology advocates pathological staging with modified inguinal lymphadenectomy or dynamic sentinel lymph node biopsy (DSNB) [2,3]. The concept of the sentinel lymph node scintigraphy is based on the principle that histopathological examination of the first draining lymph node(s) can reliably predict the status of the regional lymphatic spreading. Therefore, a negative sentinel lymph node biopsy may reliably exclude regional lymph node metastasis in appropriately selected patients. The indications and protocols for sentinel lymph node biopsy are defined by international guidelines. According to the EAU–ASCO guidelines, dynamic sentinel lymph node biopsy is recommended for clinically node-negative patients with high-risk penile cancer (T1b or higher) [4].

Early detection and removal of metastases improve survival compared with delayed lymphadenectomy performed only after clinically evident lymph node involvement develops [5]. The most important goals of treatment are to avoid unnecessary morbidity while ensuring the reliable detection of micrometastases.

Since the original concept of the sentinel lymph node (SNL) proposed by Cabañas [6], SNL biopsy has been widely adopted, although with variable results [7,8]. The aim of our study was to evaluate the reliability, accuracy, and morbidity of DSNB in the management of intermediate- and high-risk penile cancer patients with clinically negative lymph node status during long-term follow-up.

## 2. Materials and Methods

### 2.1. Study Design and Patient Population

A total of 287 patients with histologically proven penile cancer underwent radioisotope-guided DSNB between January 2005 and January 2024 at the Department of Urology, Medical School, University of Pécs, Hungary. Thirty-nine patients were lost to follow-up within the first 24 months after the procedure and were therefore excluded from the study. Of the remaining 248 patients, 211 underwent primary DSNB (performed simultaneously with primary tumour treatment), while 37 underwent secondary DSNB (performed during a second session after primary tumour treatment).

**Figure 1:**
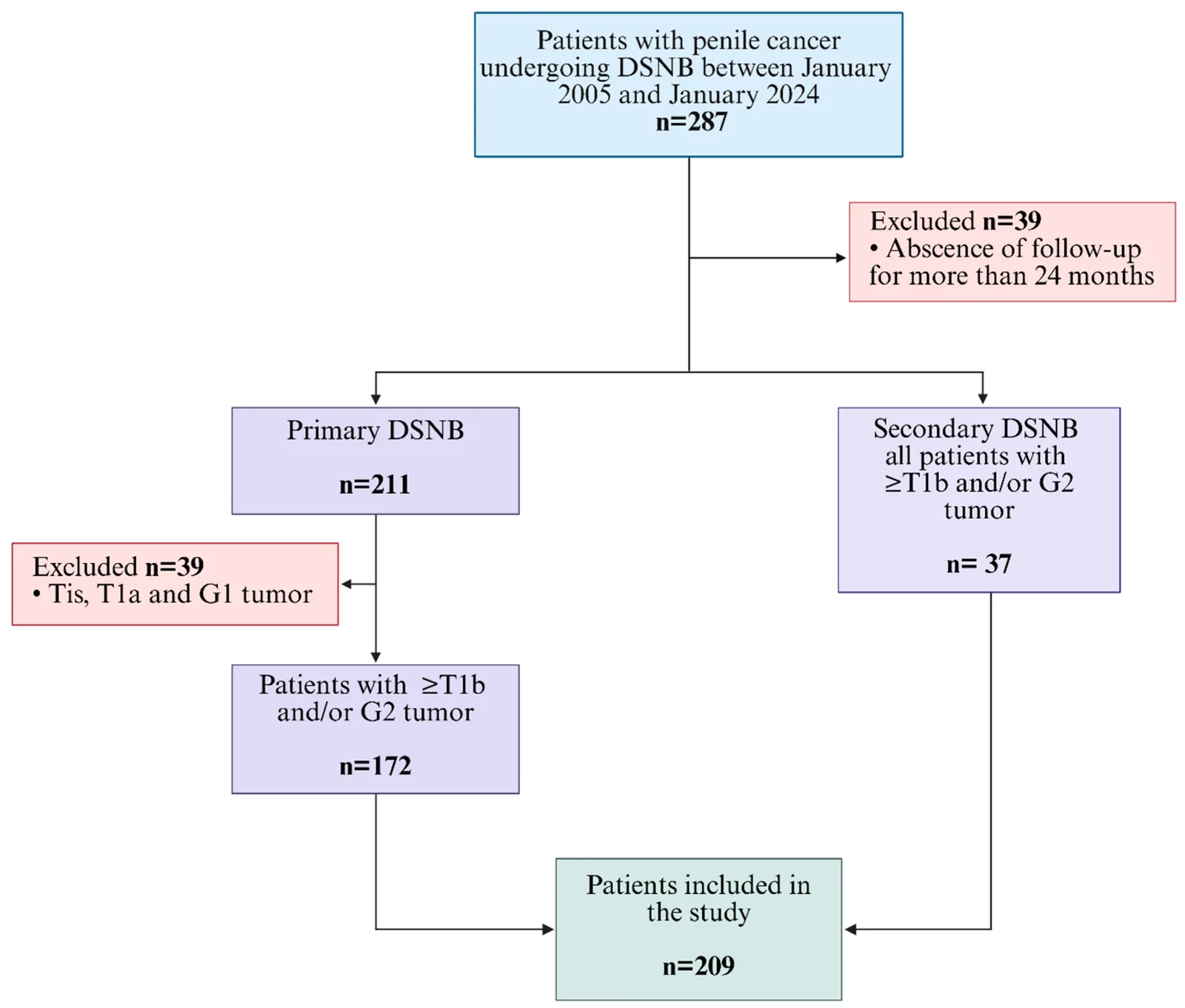
Flowchart of patient inclusion criteria. Created in https://BioRender.com.

All patients who underwent secondary DSNB had a primary tumour histology of ≥T1b and/or G2. In the primary DSNB group, 39 patients with histologically confirmed Tis, T1a, or G1 primary tumours were also excluded from the study. After these exclusions, a total of 209 patients were eligible for analysis (Figure 1). The study was reviewed and approved by the Regional Research Ethics Committee of the University of Pécs (91-PTERKEB2026/1).

### 2.2. Preoperative Assessment

As part of the preoperative diagnostic workup, all patients underwent physical examination and inguinal ultrasonography. Based on negative findings, patients were classified as having clinically node-negative penile cancer. In the primary DSNB group, primary tumour treatment (all procedures were penile-sparing surgeries) was performed, and all patients underwent lymphoscintigraphy after providing written informed consent.

### 2.3. Radiotracer Preparation and Administration

For SLN scintigraphy, human serum albumin millimicroaggregate, 1.0 mg per vial (Senti-Scint, Medi-Radiopharma Ltd., Érd, Hungary), was used. The radiotracer was prepared according to the manufacturer’s instructions. Depending on the daily patient workload, approximately 0.5–1.0 GBq of freshly eluted sterile sodium pertechnetate (99mTc-pertechnetate) in a volume of 3 mL was added to the lyophilized kit. The lyophilized material was dissolved by gentle swirling and incubated at room temperature for 20 min. During incubation, the vial was gently swirled intermittently to ensure homogeneous labelling. Following incubation, sterile saline was added to facilitate dose dispensing. Individual patient doses of 100 MBq in a total volume of 1 mL were drawn into 2 mL Luer-lock syringes. A 0.5 × 25 mm needle was attached for administration.

**Figure 2:**
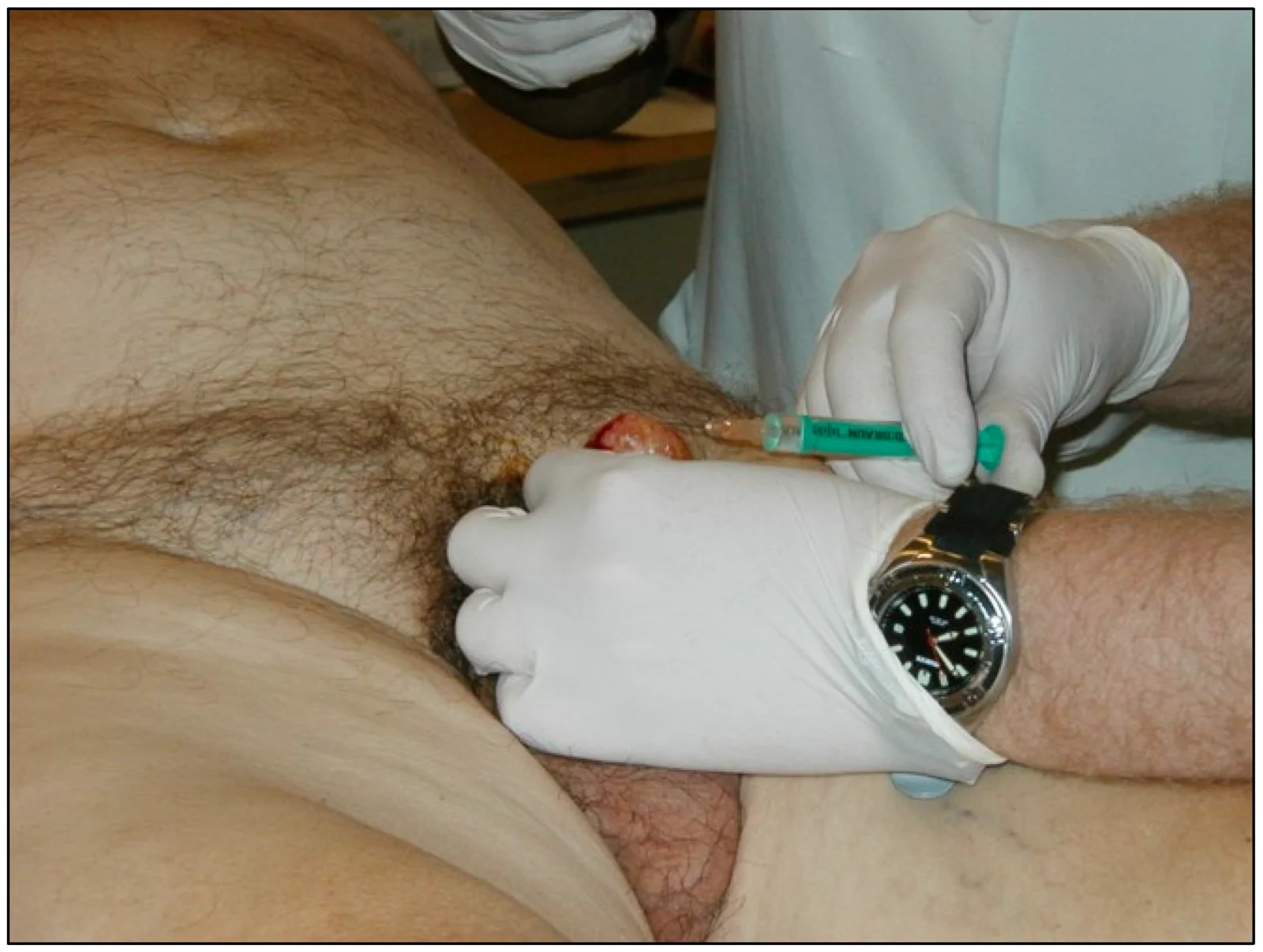
Representative image of administration of radiotracer. Own image.

In the primary setting, the radiotracer was administered peritoumorally and subcutaneously at four separate injection sites surrounding the lesion. In cases where the tumour involved the entire glans, the injection was performed at the penile shaft. Penile shaft injections were also employed in the secondary DSNB setting (Figure 2).

### 2.4. Lymphoscintigraphy

**Figure 3:**
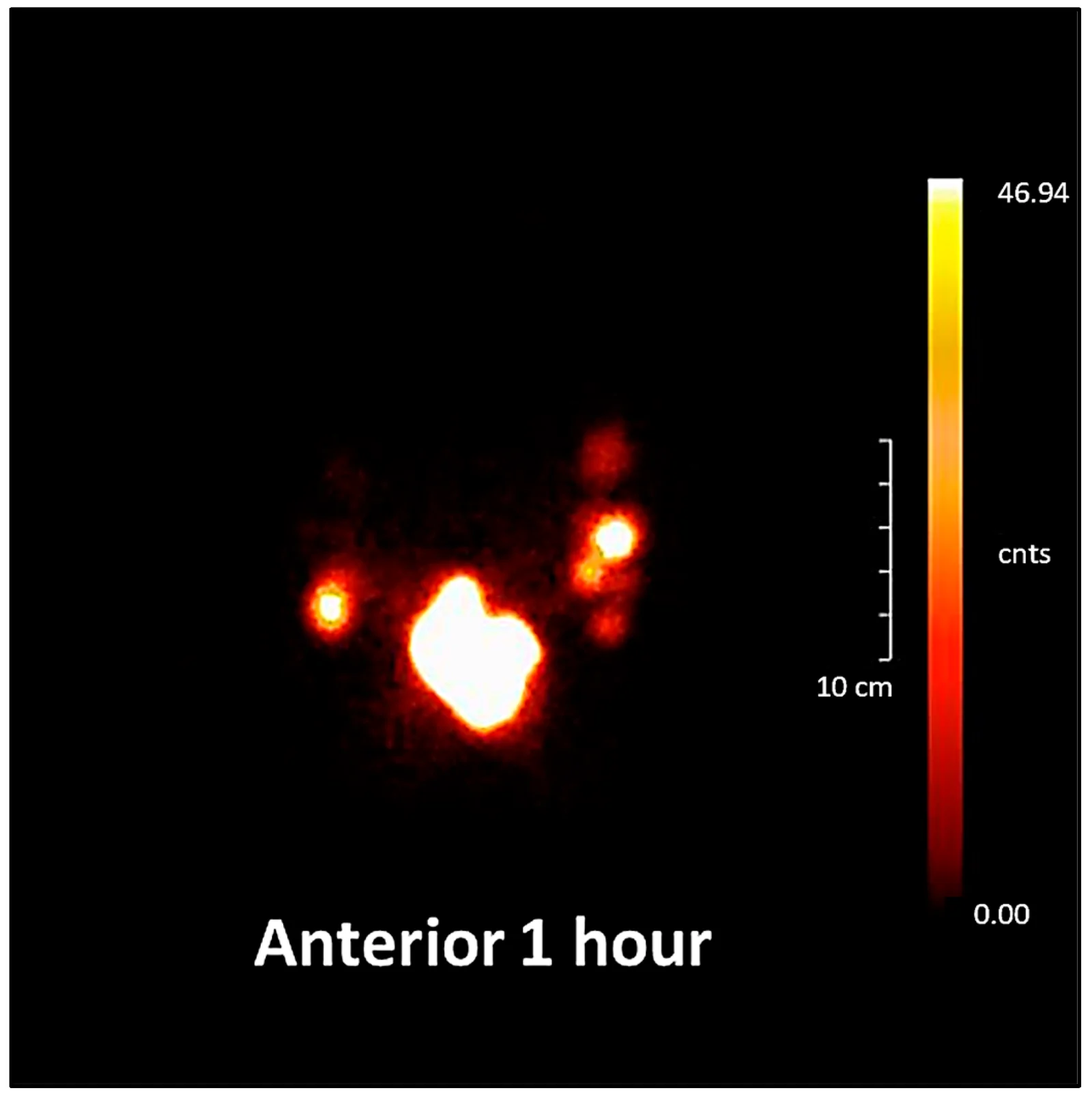
Summed images (anterior view) at 1 h following penile shaft injection of 99mTc-nanocolloid. Multiple SLN-s bilaterally. Own image.

Planar anterior images were acquired immediately after tracer administration (early phase), as well as at 1 and 3 h after injection, using a single-head gamma camera (MB9200, Mediso Ltd., Budapest, Hungary) equipped with a low-energy general-purpose (LEGP) collimator. Acquisition parameters included a 256 × 256 matrix, with imaging continued until 500,000 counts had been collected (Figure 3).

### 2.5. Dynamic Sentinel Lymph Node Biopsy

**Figure 4:**
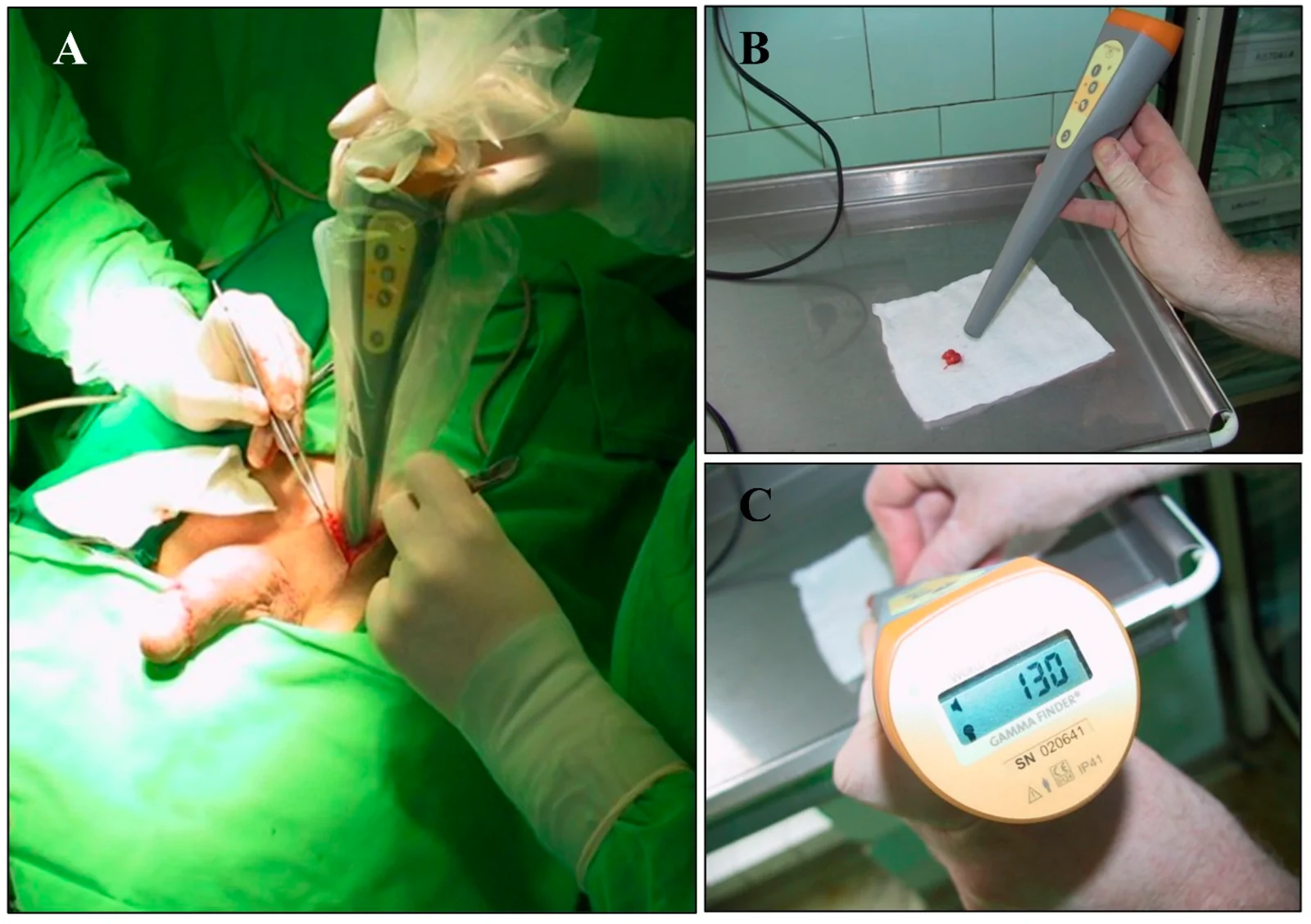
Dynamic Sentinel Lymph Node Biopsy. Intraoperative use of Gamma Finder II (**A**) and ex vivo analysis of fresh biopsy sample (**B**,**C**). Own images.

Based on the scintigraphic findings, the location of the identified SLN(s) was marked on the skin surface using a permanent marker. Intraoperative localization was carried out using an intraoperative gamma probe. The vast majority of DSNB procedures were performed one day after lymphoscintigraphy (two-day protocol); in three cases, lymph node dissection was performed on the same day as lymphoscintigraphy (one-day protocol). For all procedures, a Gamma Finder II probe (W.O.M. WORLD OF MEDICINE GmbH, Reichenbach, Germany) was used (Figure 4).

All procedures were performed by a single surgeon. All identified isotopically active lymph nodes were dissected and sent for histological examination.

### 2.6. Histopathological Processing and Evalutation

All excised lymph nodes were fixed in 10% neutral-buffered formalin and subsequently embedded in paraffin. Five-µm-thick tissue sections were prepared and deparaffinized in xylene, followed by rehydration through a descending series of ethanol solutions (97%, 80%, 70%, and 50%) and distilled water. Sections were then stained with Mayer’s hematoxylin solution (Sigma-Aldrich, St. Louis, MO, USA) for 10 min and subsequently washed. Following hematoxylin staining, the sections were exposed to 0.25% acetic acid and stained with eosin solution (Sigma-Aldrich, St. Louis, MO, USA) for 2 min. Finally, the sections were dehydrated through an ascending series of ethanol solutions, cleared in xylene, and mounted using DPX mounting medium. Representative images were digitized with Mirax scanner and photographs were taken with CaseViewer 2.4 software (3DHISTECH Ltd., Budapest, Hungary). Histopathological evaluation was performed by a single trained and experienced uropathologist.

### 2.7. Follow-Up

**Figure 5:**
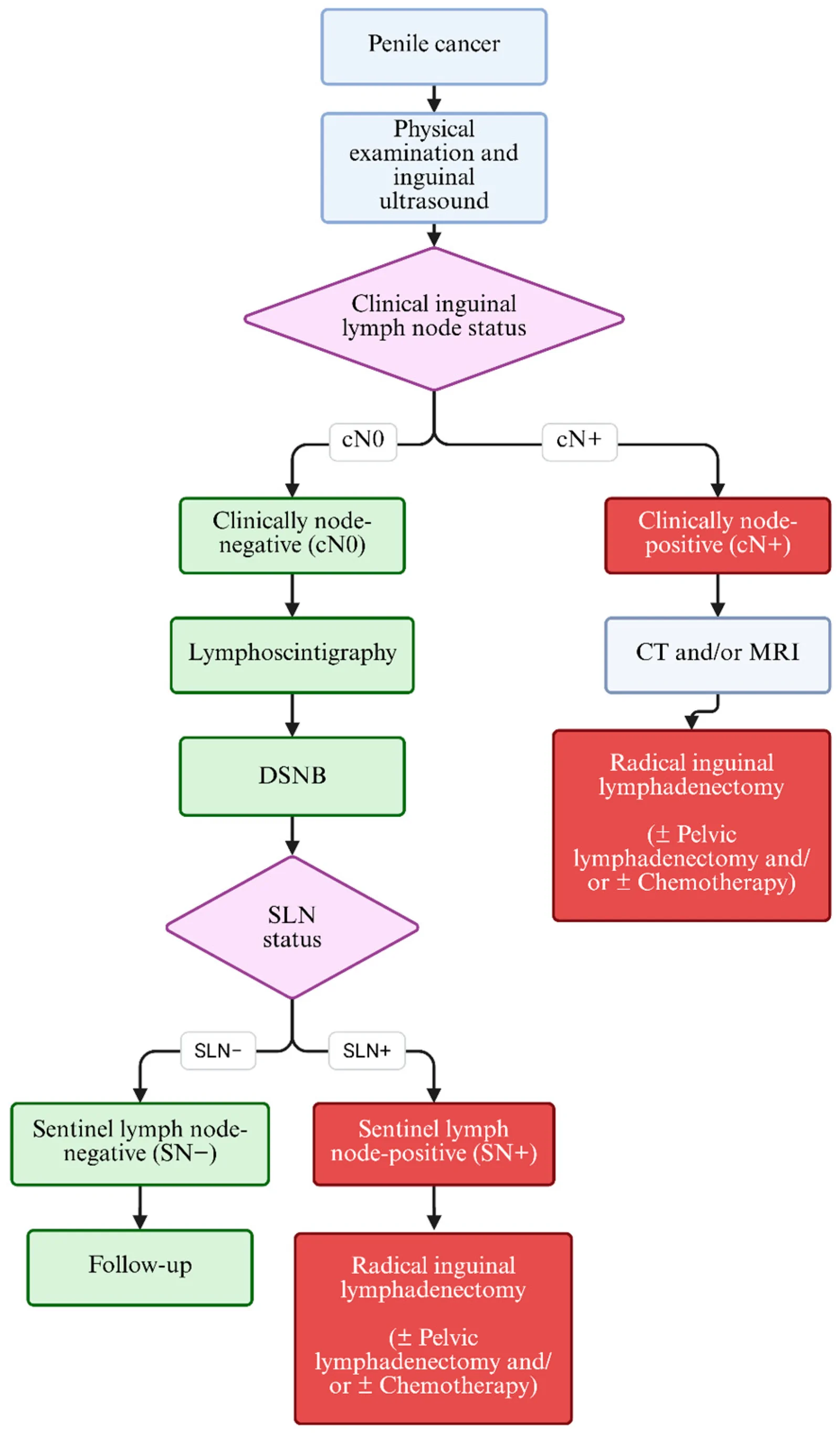
Flowchart of patient routes. Created in https://BioRender.com.

All patients with positive SLN status underwent radical inguinal lymphadenectomy. Patients with negative SLN status were enrolled in a follow-up protocol consisting of physical examination and inguinal ultrasonography every 3 months, together with annual MRI examinations (Figure 5).

### 2.8. Statistical Analysis

The collected variables included primary tumour stage and grade [2], number of SLNs, SLN histology, DSNB-related adverse events, progression to inguinal metastasis, false-negative rate of the procedure, and, when performed, histological findings from radical lymphadenectomy.

The number of SLNs per patient was summarized using the mean and interquartile range (IQR), calculated as Q3 − Q1, where Q3 and Q1 represent the 75th and 25th percentiles, respectively.

Sensitivity was calculated using the following formula: sensitivity = true-positive procedures/(true-positive procedures + false-negative procedures). DSNB was considered false negative when all removed SLNs were histologically negative and the patient subsequently developed regional inguinal recurrence in the absence of a new primary tumour or local tumour recurrence. The false-negative rate was calculated using the following formula: False-negative rate = false-negative procedures/(true-positive procedures + false-negative procedures). Exact 95% confidence intervals (CIs) for sensitivity and false-negative rates were calculated using the Clopper–Pearson method.

## 3. Results

Our study included 209 patients, in whom a total of 573 SLN were identified and removed, resulting in an average number of 2.7 SLNs per patient (IQR: 8). An interesting change in the number of SLNs could be observed depending on the radiotracer injection site. In the 163 patients in the primary DSNB group who received peritumoral tracer injections, the average number of SLNs was 2.3 (IQR: 5). In the 9 patients who underwent primary DSNB with penile shaft tracer injection, the mean of SLNs was 4.2 (IQR: 5), while in the 37 patients who underwent secondary DSNB with penile shaft tracer injection, the average number of detected SLNs was 4.3 (IQR: 7) (Table 1).

**Figure 6:**
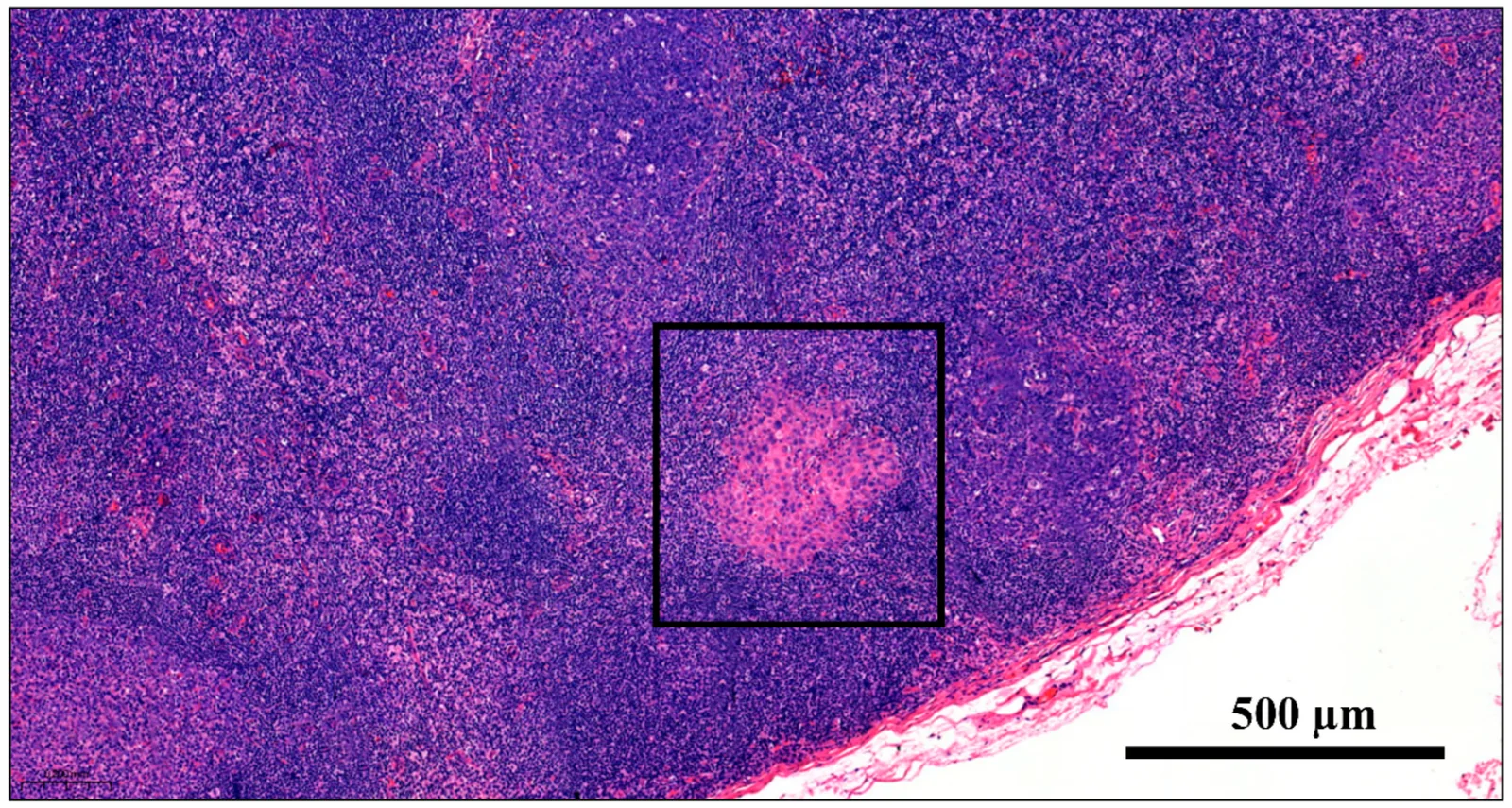
Representative image of HE stained metastatic SLN. Micrometastasis highlighted in black rectangle. Scale: 500 µm Own image.

Histological examination revealed SLN metastases in 31 patients (Figure 6) corresponding to a total of 37 metastatic groins. All patients with histologically proven metastases underwent ipsilateral radical inguinal lymphadenectomy in a second procedure (or bilateral lymphadenectomy in cases of bilateral SLN positivity). In addition to the positive lymph nodes already removed during DSNB, further lymph node involvement was detected in 6 groins in 5 patients (Table 2). All sentinel lymph node metastases were macrometastases, defined as tumor deposits exceeding 2 mm in greatest dimension. In all cases, the metastatic involvement was confined to the lymph node, and no extracapsular extension was observed. All patients were classified as having pN1 disease on final pathological assessment; therefore, no adjuvant therapy was administered. No distant metastases, local recurrence, or inguinal recurrence were observed during the follow-up period.

In the remaining 26 patients, histological examination of the radical inguinal lymphadenectomy specimens revealed no additional metastases.

Patients with negative SLN status underwent a rigorous follow-up protocol, including physical examination and inguinal ultrasound every 3 months, as well as annual inguinal and abdominopelvic MRI and low-dose native chest CT. After 5 years of follow-up, the surveillance schedule was reduced to visits every 6 months. In the case of suspicious findings on physical examination or ultrasound, immediate MRI and fine-needle aspiration biopsy (FNAB) of the suspected lymph nodes were performed. If FNAB confirmed metastasis, ipsilateral inguinal lymphadenectomy was carried out. Otherwise, the follow-up protocol continued as described above, and lymphadenectomy was performed if further progression was detected on MRI despite a negative FNAB result. All 209 patients had a minimum follow-up period of 2 years, and in 118 cases the follow-up exceeded 5 years. The median follow-up time was 38 months (IQR: 103 months).

Among the 178 patients with negative SLN status, three developed inguinal metastases during the follow-up period. None of these patients had evidence of a newly diagnosed primary tumour or local recurrence at the primary tumour site; therefore, these cases were classified as false-negative results. The sensitivity of DSNB was 91.2% (95% CI: 76.3–98.1%) on a per-patient basis and 92.5% (95% CI: 79.6–98.4%) on a per-groin basis. The corresponding false-negative rates were 8.8% (95% CI: 1.9–23.7%) and 7.5% (95% CI: 1.6–20.4%), respectively.

All false-negative groins were detected within the first 2 years of follow-up, and no further recurrences were observed thereafter. Three nodal metastases were detected during follow-up: one at 6 months and two at 9 months after the initial procedure. All three patients underwent inguinal lymph node dissection. Final histopathological staging showed pN1 disease in two patients and pN3 disease in one patient, owing to extranodal extension. No adjuvant treatment was administered to the patients with pN1 disease, and no further progression was observed during follow-up. The patient with pN3 disease received adjuvant chemotherapy; however, he subsequently died of causes unrelated to penile cancer.

Overall, 24 of the 209 patients (11.4%) experienced complications following DSNB. Most complications were mild or moderate and could be managed on an outpatient basis. Only two patients required surgical intervention, and one patient required hospital readmission. The complications are summarized in Table 3.

## 4. Discussion

Controversy persists regarding the management of clinically negative lymph node patients with penile cancer. Surveillance protocols carry the risk of missing subsequent micrometastases [5,9], while inguinal lymphadenectomy is associated with high morbidity and is necessary in only 20–25% of cases [10,11]. To balance oncological safety with the need to avoid unnecessary overtreatment and surgery-related morbidity, the EAU Guidelines advocate invasive lymph node staging using modified lymphadenectomy or DSNB [2].

Since Horenblas et al. and Wawroschek et al. described the technique of SLN biopsy in penile cancer, the procedure has been widely used [12,13]. In this retrospective study, we investigated the reliability, accuracy, and complications of isotope-guided DSNB during long-term follow-up. The data were obtained from a tertiary supraregional referral centre for penile cancer, with more than two decades of experience in the gamma probe-guided SLN biopsy technique [14]. To our knowledge, this study presents data from the largest series of patients treated with DSNB in Central Europe.

In the present study, we demonstrated a sensitivity rate of 91% per patient and 92.5% per groin. The false-negative rates were 8% per patient and 7.5% per groin, respectively, while the negative predictive value was 98%. The overall complication rate was 11.4%. The vast majority of complications were manageable on an outpatient basis and were mild to moderate in severity. Only two patients experienced complications greater than Clavien–Dindo [15] Grade II (0.9%).

Two large meta-analyses evaluating the reliability of DSNB demonstrated weighted sensitivity rates of 87% and 88%, respectively, and both studies reported a wide range of sensitivities (50–98%) [16,17]. Meta-regression analysis revealed that institutional experience with DSNB was significantly associated with heterogeneity among sensitivity rates. Procedures performed in centres with experience of more than 30 cases showed relatively stable results. Our findings support these observations, demonstrating a higher sensitivity rate than the weighted sensitivity reported in these meta-analyses and results comparable to those reported by high-volume centres.

Published complication rates for the procedure vary considerably, ranging from 4.5% to 27%, with most complications being mild or moderate [18,19,20,21]. Our overall complication rate of 11.4% is highly comparable to that reported in other large series.

The integration of molecular biomarkers, including microRNA profiling, may improve risk stratification and the prediction of metastatic disease, thereby enabling more appropriate patient selection, reducing the need for unnecessary pathological staging, and minimizing the risk of procedure-related complications [22].

An interesting observation was made regarding the total number of detected SLNs according to the radiotracer injection site. When the injection was performed peritoumorally, the average number of detected SLNs was 2.3 (IQR: 5). In contrast, with penile shaft injection at the primary procedure, the average number was 4.2 (IQR: 5), and 4.3 (IQR: 7) at the secondary procedure. We could not identify published data directly comparing these approaches. Although the injection site and the number of detected SLN do not appear to influence sensitivity and reliability, they may be associated with higher complication rates. The only patient in our cohort who required reoperation had nine SLNs identified; however, the small patient group is insufficient for meaningful statistical analysis of this observation.

Because this study encompasses data collected over a long time frame, the two-day protocol was employed in the vast majority of cases, reflecting the routine practice at our institution as well as at many other centres. Recently Dimopoulos et al. reported comparative data for one-day and two-day procedures and advocated the one-day approach because of a higher number of detected SLNs and a lower complication rate [21]. To our knowledge this is the only comparative study of the one-day, and two-day protocol. In our series, we also performed the one-day procedure in three cases. However, we did not observe any differences compared with the two-day procedure, although the number of cases is too small to allow meaningful analysis.

The most widely used technique in Europe is based on 99mTc-labelled albumin nanocolloids. In addition, receptor-mediated tracers such as 99mTc-tilmanocept have been investigated for SLN testing in other cancers, like breast cancer and oral cancer. Tilmanocept binds to the mannose receptor (CD206) expressed on macrophages and dendritic cells, providing high, specific retention in the SLN and less accumulation in distal nodes [23]. In the future, the application of this tracer in penile cancer and its comparison with albumin-based nanocolloids or other techniques may be of particular interest. Different approaches are available for such comparisons, including hybrid tracers combining 99mTc-labelled nanocolloids with indocyanine green (ICG), as well as other fluorescence and magnetic-based techniques using superparamagnetic iron oxide nanoparticles/SPIONs. Although these novel approaches appear promising, their only proven advantage is enhanced visualisation and superiority compared with blue dye. However, no superiority over the conventional radiotracer has been demonstrated. Moreover, there is currently insufficient evidence to show a statistically significant improvement in either sensitivity or false-negative rates when hybrid tracer techniques are employed [24,25,26].

The main limitation of our study is its retrospective design, whereas its strengths are the large patient cohort and the long-term follow-up.

## 5. Conclusions

Our results suggest that, in a high-volume centre, DSNB is a safe and reliable treatment option with low morbidity for patients with intermediate- and high-risk penile cancer and clinically negative lymph node status. Further studies and analyses are needed to determine the importance of radiotracer injection sites and to compare one-day and two-day procedures.

## Figures and Tables

**Table 1:** Number of sentinel lymph nodes found.

	Primary DSNB, Peritumoral Injection (N = 163)	Primary DSNB Penile Shaft Injection (N = 9)	Secondary DSNB Penile Shaft Injection (N = 37)
**Total Number of SLN**	376	38	159
**Average Number of SLN (IQR)**	2.3 (5)	4.2 (5)	4.3 (7)

**Table 2:** Tumor stage and histopathological results of DSNB.

Tumor Stage	DSNB pN0 (n = 178)	DSNB pN+ (n = 31)	RILND pN+ (n = 5)
**pT1G2**	68	3	0
**pT1G3**	61	6	1
**pT2G2**	18	9	2
**pT2G3**	31	13	2

**Table 3:** Summary of 24 complications related to the 209 DSNB procedures.

Complication	Number of Patients n (%)	Clavien–Dindo Classification Grade
Seroma/Lymphocele requiring no intervention	6 (2.8%)	I
Wound dehiscence requiring conservative wound care	5 (2.3%)	I
Hematoma requiring no intervention	4 (1.9%)	I
Leg oedema requiring compression stockings	4 (1.9%)	I
Wound infection requiring antibiotic treatment	3 (1.4%)	II
Lymphocele requiring ultrasound-guided drainage	1 (0.4%)	IIIa
Wound dehiscence requiring surgical revision	1 (0.4%)	IIIb
**Total**	**24 (11.4%)**	

## Data Availability

The data sets used and/or analysed during the current study are available from the corresponding author on reasonable request.

## Abbreviations

DSNB	dynamic sentinel lymph node biopsy
FNAB	fine-needle aspiration biopsy
IQR	interquartile range
LEGP	low-energy general purpose
SLN	sentinel lymph node

## References

[1] Cardona C.E.M., García-Perdomo H.A. (2018). Incidence of penile cancer worldwide: Systematic review and meta-analysis. Rev. Panam. Salud Pública.

[2] Parnham A., Albersen M., Ayres B., Crook J., Johnstone P.A., Oliveira P., Pagliaro L.C., Pettaway C., Protzel C., Rumble R.B. (2026). European Association of Urology and American Society of Clinical Oncology Guidelines on Penile Cancer: A Summary of the 2026 Guidelines Update. Eur. Urol..

[3] Ficarra V., Akduman B., Bouchot O., Palou J., Tobias-Machado M. (2010). Prognostic factors in penile cancer. Urology.

[4] Brouwer O.R., Rumble R.B., Ayres B., Sánchez Martínez D.F., Oliveira P., Spiess P.E., Johnstone P.A.S., Crook J., Pettaway C.A., Tagawa S.T. (2024). Penile Cancer: EAU-ASCO Collaborative Guidelines Update Q and A. JCO Oncol. Pract..

[5] Kroon B., Horenblas S., Lont A., Tanis P., Gallee M., Nieweg O. (2005). Patients with penile carcinoma benefit from immediate resection of clinically occult lymph node metastases. J. Urol..

[6] Cabanas R.M. (1977). An approach for the treatment of penile carcinoma. Cancer.

[7] Vanthoor J., Thomas A., Tsaur I., Albersen M., European Reference Network for Rare Urogenital Diseases and Complex Conditions (eUROGEN) (2020). Making surgery safer by centralization of care: Impact of case load in penile cancer. World J. Urol..

[8] Bloom J.B., Stern M., Patel N.H., Zhang M., Phillips J.L. (2018). Detection of lymph node metastases in penile cancer. Transl. Androl. Urol..

[9] McDougal W.S. (1995). Carcinoma of penis: Improved survival by early regional lymphadenectomy based on histological grade and depth of invasion of primary lesion. J. Urol..

[10] Bevan-Thomas R., Slaton J.W., Pettaway C.A. (2002). Contemporary morbidity from lymphadenectomy for penile squamous cell carcinoma: The MD Anderson Cancer Center Experience. J. Urol..

[11] Stuiver M.M., Djajadiningrat R.S., Graafland N.M., Vincent A.D., Lucas C., Horenblas S. (2013). Early wound complications after inguinal lymphadenectomy in penile cancer: A historical cohort study and risk-factor analysis. Eur. Urol..

[12] Horenblas S., Jansen L., Meinhardt W., Hoefnagel C.A., de Jong D., Nieweg O.E. (2000). Detection of occult metastasis in squamous cell carcinoma of the penis using a dynamic sentinel node procedure. J. Urol..

[13] Wawroschek F., Vogt H., Bachter D., Weckermann D., Hamm M., Harzmann R. (2000). First experience with gamma probe guided sentinel lymph node surgery in penile cancer. Urol. Res..

[14] Pytel A., Damasdi M., Schmidt E., Frick A., Farkas L. (2011). 975 Advantages of Dynamic Sentinel Lymphnode Biopsy Technique in the Management of Penile Cancer. J. Urol..

[15] Dindo D., Cuesta M.A., Bonjer H.J. (2014). The Clavien–Dindo Classification of Surgical Complications. Treatment of Postoperative Complications After Digestive Surgery.

[16] Fallara G., Pozzi E., Cakir O.O., Tandogdu Z., Castiglione F., Salonia A., Alnajjar H.M., Muneer A. (2023). Diagnostic accuracy of dynamic sentinel lymph node biopsy for penile cancer: A systematic review and meta-analysis. Eur. Urol. Focus.

[17] Zou Z.-J., Liu Z.-H., Tang L.-Y., Wang Y.-J., Liang J.-Y., Zhang R.-C., Tang Y.-Q., Lu Y.-P. (2016). Radiocolloid-based dynamic sentinel lymph node biopsy in penile cancer with clinically negative inguinal lymph node: An updated systematic review and meta-analysis. Int. Urol. Nephrol..

[18] Leijte J.A., Hughes B., Graafland N.M., Kroon B.K., Olmos R.A.V., Nieweg O.E., Corbishley C., Heenan S., Watkin N., Horenblas S. (2009). Two-center evaluation of dynamic sentinel node biopsy for squamous cell carcinoma of the penis. J. Clin. Oncol..

[19] Lam W., Alnajjar H.M., La-Touche S., Perry M., Sharma D., Corbishley C., Pilcher J., Heenan S., Watkin N. (2013). Dynamic sentinel lymph node biopsy in patients with invasive squamous cell carcinoma of the penis: A prospective study of the long-term outcome of 500 inguinal basins assessed at a single institution. Eur. Urol..

[20] Nemitz L., Vincke A., Michalik B., Engels S., Meyer L.-M., Henke R.-P., Wawroschek F., Winter A. (2022). Radioisotope-guided sentinel lymph node biopsy in penile cancer: A long-term follow-up study. Front. Oncol..

[21] Dimopoulos P., Christopoulos P., Shilito S., Gall Z., Murby B., Ashworth D., Taylor B., Carrington B., Shanks J., Clarke N. (2016). Dynamic sentinel lymph node biopsy for penile cancer: A comparison between 1-and 2-day protocols. BJU Int..

[22] Murta C.B., Furuya T.K., Carrasco A.G.M., Uno M., Sichero L., Villa L.L., Faraj S.F., Coelho R.F., Guglielmetti G.B., Cordeiro M.D. (2022). miRNA and mRNA Expression Profiles Associated with Lymph Node Metastasis and Prognosis in Penile Carcinoma. Int. J. Mol. Sci..

[23] Rovera G., de Koster E.J., Rufini V., Zollino M., Zagaria L., Giammarile F., Vidal-Sicart S., Valdés Olmos R., Collarino A. (2023). (99m)Tc-Tilmanocept performance for sentinel node mapping in breast cancer, melanoma, and head and neck cancer: A systematic review and meta-analysis from a European expert panel. Eur. J. Nucl. Med. Mol. Imaging.

[24] Dell’Oglio P., de Vries H.M., Mazzone E., KleinJan G.H., Donswijk M.L., van der Poel H.G., Horenblas S., van Leeuwen F.W.B., Brouwer O.R. (2020). Hybrid Indocyanine Green-(99m)Tc-nanocolloid for Single-photon Emission Computed Tomography and Combined Radio- and Fluorescence-guided Sentinel Node Biopsy in Penile Cancer: Results of 740 Inguinal Basins Assessed at a Single Institution. Eur. Urol..

[25] Torbrand C., Warnolf Å., Glombik D., Davidsson S., Carlsson J., Baseckas G., Håkansson U., Trägårdh E., Geijer H., Liedberg F. (2022). Sentinel Node Identification with Hybrid Tracer-guided and Conventional Dynamic Sentinel Node Biopsy in Penile Cancer: A Prospective Study in 130 Patients from the Two National Referral Centres in Sweden. Eur. Urol. Oncol..

[26] Michalik B., Engels S., Otterbach M.C., Maurer M.H., Wawroschek F., Winter A. (2025). Bimodal inguinal sentinel lymph node imaging using a radiation-free fluorescent magnetic hybrid tracer in penile cancer patients. Front. Oncol..

